# Targeting Mutant *KRAS* in Pancreatic Cancer: Futile or Promising?

**DOI:** 10.3390/biomedicines8080281

**Published:** 2020-08-11

**Authors:** Friederike Inga Nollmann, Dietrich Alexander Ruess

**Affiliations:** Department of General and Visceral Surgery, Center of Surgery, Medical Center–University of Freiburg, Hugstetterstrasse 55, 79106 Freiburg, Germany; friederike.nollmann@uniklinik-freiburg.de

**Keywords:** pancreatic cancer, KRAS mutation, treatment options

## Abstract

Pancreatic ductal adenocarcinoma (PDAC) is one of the most fatal cancers with a dismal prognosis for the patient. This is due to limited diagnostic options for the early detection of the disease as well as its rather aggressive nature. Despite major advances in oncologic research in general, the treatment options in the clinic for PDAC have only undergone minor changes in the last decades. One major treatment advance would be the successful targeting of the oncogenic driver *KRAS^mut^*. In the past, the indirect targeting of KRAS has been exploited, e. g., via upstream inhibition of receptor tyrosine kinases or via downstream MEK or PI3K inhibition. However, the experience gained from clinical trials and from the clinic itself in the treatment of KRAS^mut^ cancer entities has dampened the initial euphoria. Lately, with the development of KRAS^G12C^-specific inhibitors, not only the direct but also the indirect targeting of KRAS^mut^ has gained momentum again. Though preclinical studies and preliminary early clinical studies of monotherapies have shown promising results, they have been overshadowed by the swift development of resistances resulting in inconsistent responses in patient cohorts. Currently, several different combination therapies for KRAS^mut^ cancer are being explored. If they hold the promise they have made in preclinical studies, they might also be suitable treatment options for patients suffering from PDAC.

## 1. Introduction

Pancreatic ductal adenocarcinoma (PDAC) remains one of the most devastating of all cancer types with a 5-year survival rate of less than 10% [1,2]. Due to progress in the treatment of other cancer entities, such as breast cancer or lung adenocarcinoma, PDAC is predicted to become one of the leading causes of cancer-related deaths by 2030 worldwide [3,4]. Over the past decades, only minor changes in its management and treatment were implemented in the clinic to improve patients’ prognosis, at the cost of increased toxicity [5]. Surgical resection remains the only potentially curative treatment option, but merely 20% of patients present with the up-front resectable disease. Currently, eligible patients receive surgery and then adjuvant treatment with gemcitabine, gemcitabine/capecitabine, gemcitabine/Nab-Paclitaxel, (modified) FOLFIRINOX, or combination therapy (including other treatment modalities, such as radiotherapy), depending on their performance status [6,7,8]. The therapeutic success of clinically available first-line systemic therapies for locally advanced and metastatic disease, which are largely similar to the regimens used in the adjuvant setting, is highly limited by intrinsic and acquired chemoresistance. While promising attempts have been made to elucidate the underlying mechanisms, they still remain only poorly understood [9,10,11,12,13,14,15,16,17]. In search of additional treatment options, molecularly tailored approaches are being evaluated. Findings in (pre-) clinical studies and meta-analyses of available data suggest hope for targeted therapies as a promising way for treating PDAC [18,19]. Unfortunately, the initial high hopes set on the first targeted therapy, erlotinib, an epidermal growth factor receptor (EGFR) tyrosine kinase inhibitor (TKI), were disappointed, because of quickly developing resistance and marginal as well as mixed treatment response [20,21]. Recent preclinical findings have shed light on the involved mechanisms and suggest successful application of the TKI in vertical combination with downstream effectors, e.g., STAT3i, PI3Ki or MEKi [22,23,24]. Nevertheless, taking all of the above into consideration, the clinical development of novel treatments for patients suffering from PDAC is desperately needed. Although PDAC is characterized by a highly divers inter- and intra-tumoral mutational landscape, over 90% of all patients display an activating mutation in the *KRAS* gene [25,26,27]. Hence, mutant *KRAS* could be a valuable target in the treatment of PDAC.

## 2. KRAS

*KRAS* encodes a small GTPase, which cycles between active and inactive state upon binding of GTP, mediated by guanine nucleotide exchange factors (GEFs), and hydrolysis to GDP, facilitated by GTPase-activating proteins (GAPs), respectively. A mutation in *KRAS* mostly involves a downregulation of its intrinsic GTPase activity as well as a subdued interaction potential of KRAS with GAPs. This results in a constitutive activation of the KRAS protein and continued stimulation of downstream signaling pathways, which, in turn, govern several hallmarks of cancer, e.g., proliferation, anti-apoptosis, cell migration and metastasis [28,29]. The proto-oncogene *KRAS* is the most frequently mutated among the four main driver oncogenes in PDAC (next to *TP53*, *CDKN2A* and *SMAD4*). Even more so, it is considered the initiating genetic event in the stepwise progression from metaplasia towards malignant disease [30,31]. Though somatic mutations of *KRAS* occur in more than 90% cases of PDAC patients, its signaling cascades may also be hyperactivated by amplification of the wildtype isoform [32] or other molecular alterations in the receptor tyrosine kinase-RAS-RAF-MAPK pathway [33].

Until recently, KRAS was considered a valuable but yet undruggable target. Due to continuous research efforts over the past decades, more insight into the function and potential roles of this onco-protein was gained, leading to promising direct and indirect targeting approaches [34,35,36]. In the past, the inhibition of the protein’s farnesylation, which is essential for the immobilization at the cell membrane and subsequently for the activity of KRAS, was investigated [37]. This approach has been mostly abandoned, as no significant benefit was identified in clinical studies [38,39]. Another avenue that was explored was the suppression of KRAS expression by the means of RNA interference (RNAi) [40,41,42,43,44,45,46]. Although this approach yielded promising results not only in preclinical rodent models but also in a clinical phase I/II a study [47], the major drawbacks in terms of stability, delivery and specificity of the siRNA led to a hold in the development. Current clinical research focusses mainly on direct targeting of KRAS by binding inhibitors or indirect targeting by inhibition of upstream regulators or downstream signaling pathways as well as immune modulatory approaches.

## 3. Direct Targeting

**Figure 1:**
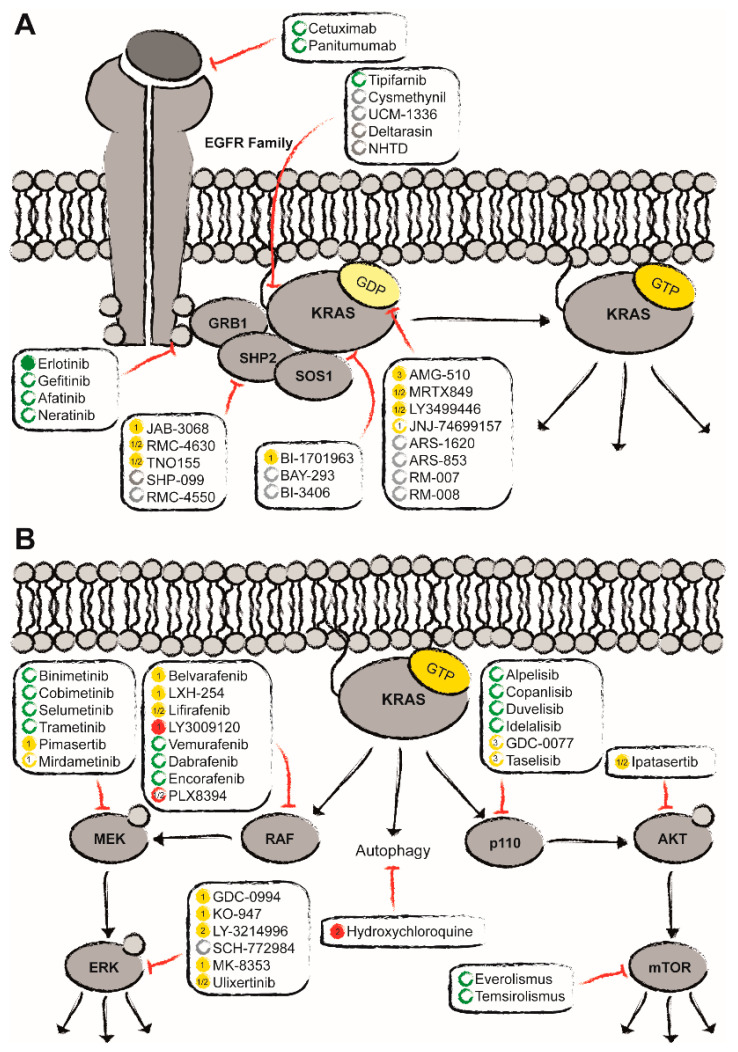
Clinical status quo of the potential treatment options for KRAS^mut^ pancreatic ductal adenocarcinoma (PDAC) (adapted from Moore et al.) [58]. Apart from the direct targeting of KRAS^mut^ with covalent allele-specific inhibitors as well as farnesylation inhibitors (**A**), indirect inhibition has gained more and more momentum (**B**). Here, approved drugs are depicted in green, ongoing clinical trials in yellow, and in red compounds that have failed to show benefit in clinical studies. Promising preclinical candidates are marked in grey. Empty circles represent the status of compounds that have not been approved or tested in PDAC. Filled circles indicate that these compounds have explicitly been approved for PDAC or tested at least in (advanced) solid tumors including PDAC.

The identification of the switch-II pocket in GDP–KRAS^G12C^ complexes and covalent binders thereof by Shokat et al. [48] marked a milestone in turning KRAS into a druggable target. Despite the fact that KRAS is mostly present in its active form in vivo, the covalent binding of inactive KRAS proved to be biologically efficacious (Figure 1A) [49,50]. Together with Araxes Pharma and Janssen Pharmaceutical, scientists were able to develop a promising candidate (ARS-3248/JNJ-74699157) for mutated KRAS^G12C^ and submit it to clinical testing [51]. However, Amgen, working in parallel on a similar approach, beat them to it: the company’s AMG-510, which is currently tested in a phase I/II and a pivotal phase III study in solid tumors, is the clinically most advanced KRAS^G12C^ inhibitor. Notably, it has already shown promising results in patients with colorectal cancer or lung adenocarcinoma, inducing stable disease or even partial response [52,53]. In depth studies in murine models revealed that AMG-510 not only induced regressive disease due to inhibiting the KRAS signaling cascades, but additionally led to long-term anti-tumor T-cell responses [53]. Another promising candidate that also exploits the covalent binding of the switch-II pocket in GDP-KRAS^G12C^ is MTRX849. Having been developed by Mirati Therapeutics Inc., the compound was able to induce a partial response after multiple lines of treatment in patients suffering from metastatic colon adenocarcinoma or stage IV lung adenocarcinoma [54]. Unfortunately, the most prevalent point mutations of the oncogene found in PDAC result in KRAS^G12V^, KRAS^G12D^, KRAS^G12R^ and KRAS^Q61H^. KRAS^G12C^ is less frequent and only occurring in 1–3% of the cases. The frequency of the individual point mutations, as well as their impact on the structural conformation of KRAS differ. Since this effects the nucleotide binding state, it is directly connected to the biological activity: the ratio of activated vs. inactivated KRAS. This ratio is controlled by nucleotide exchange and (intrinsic) hydrolysis. Generally speaking, mutations in codon 12, 13 and 61 have a largely diminished intrinsic GTPase activity (up to 40- to 80-fold lower than KRAS^wt^) [55]. Surprisingly, KRAS^G12C^ shows an intrinsic hydrolysis rate that is comparable to that of the wildtype version. The GAP affinity and therefore the GAP-stimulated hydrolysis rate is similarly low for all mutant types and reduced by more than 90% compared to wildtype KRAS. Additionally, the interaction potential with GEFs and other downstream effectors varies [55]. Consequently, all these mechanistic changes result in stabilization of the activated GTP-bound form of the protein and mutation-specific cycling times [56,57].

Researchers of Boehringer Ingelheim performed high throughput fragment-based screening followed by structure-based drug design, successfully identifying compounds binding to the switch-I/II pockets. Taking advantage of the fact that these pockets are not only present in the inactive but also in the active form of mutated KRAS as well as the wildtype protein, they were able to design compounds with IC_50_ values in a low micromolar range [59]. Noteworthy, the switch-I/II pockets are involved in the binding of GEFs (e.g., SOS1), GAPs and signaling effectors [60,61]. These findings fueled the development of the first pan-KRASi, BI-1701963, which is currently under investigation in a phase I/II study in solid tumors, including metastatic PDAC. In contrast to the currently developed allele-specific inhibitors, e.g., AMG-510, this pan-KRASi does not bind KRAS itself but rather SOS1 and thereby inhibits the interaction of SOS1 with KRAS, resulting in a stabilization of the inactive KRAS-GDP complex. This technically indirect inhibition of KRAS bears the potential of a broader applicability, but at the costs of specificity and efficacy.

Despite very promising preliminary data, it becomes more and more evident that the sole inhibition of mutant *KRAS* is succeeded by swift development of resistance resulting in inconsistent response in patient cohorts [62,63]. This development could have been anticipated by a number of preclinical analyses in PDAC models, which uncovered resistance mechanisms evading the oncogene addiction upon KRAS inhibition [64,65,66,67]. Currently, combinations with other inhibitors (e.g., MEKi, ERKi, etc.) [53,68,69] are under investigation in clinical trials and should be further analyzed even more so in molecular detail to overcome the resistance mechanisms as well as to strengthen the KRASi potential (Table 1).

## 4. Indirect Targeting

RAS proteins form an integral part of complex and highly intertwined signaling pathways. Though substantial information has been gathered on this network, a lot still remains unknown. Indirect targeting of KRAS by disrupting upstream regulators or downstream effectors has proven difficult—the availability of several different connected pathways allows bypassing of the ones that are affected by treatment and thus negate the drugs’ effects [70,71].

### 4.1. Targeted Therapies Engaging Upstream of KRAS

Transmembrane growth factor receptors, such as EGFR, are at the forefront of regulators upstream of KRAS. For quite some time, their importance for the development of PDAC was largely dismissed because of the assumption that KRAS^mut^ is independent of stimulation. However, in-depth studies in murine models revealed that an activation of EGFR in combination with a mutation in the *KRAS* gene accelerated pancreatic carcinogenesis [72,73]. Clinical studies in other tumor entities, such as metastatic colorectal and non-small cell lung cancer, have shown that the use of anti-EGFR antibodies or tyrosine kinase inhibitors improves progression free and overall survival, but only for patients lacking a *KRAS* mutation. The subset of patients bearing a *KRAS* mutation does not profit from the treatment. Nevertheless, the EGFR-targeted treatment with erlotinib (Figure 1A) in combination with gemcitabine has successfully been transferred from research to the clinic and continues to be a treatment option for PDAC patients, without having demonstrated a stratified effect based on KRAS mutational status [74]. However, its efficacy remains controversial, since only a small subset of patients benefits from it and a definite prognostic biomarker is still at large. Other EGFR-targeted treatment options, such as the monoclonal antibodies cetuximab and panitumumab, as well as the TKIs afatinib, gefitinib or neratinib, have been approved for (metastatic) non-small cell lung cancer, HER2-positive breast cancer and (metastatic) colorectal cancer bearing no KRAS mutation. During the treatment with these EGFRi, no clinical benefit was observed for the KRAS^mut^ subpopulation similar to erlotinib [58].

For the activation of RAS, different steps are required (nucleotide exchange, processing, localization at the membrane and effector binding). Interfering with any of these steps will alter the extent of RAS activation. One GEF that has shifted into focus again is SOS1. SOS1 binds to KRAS and mediates the nucleotide exchange (GDP to GTP) [75]. Initial efforts focused amongst others on the inhibition via a direct binding of SOS1, e.g., with molecules mimicking an orthosteric SOS helix. These compounds effectively bound SOS1 but displayed only low efficacy in cellular assays [76,77]. At the moment, research focuses on preventing the interaction of KRAS with SOS1. Here, the interaction of SOS1 with the switch I/II pocket on the surface of KRAS is blocked with small molecules (see above, Figure 1A). Another potential target is the non-receptor protein tyrosine phosphatase SHP2. Although its biological function remains unclear, it has been shown that SHP2 is required for the complete activation of the mitogen-activated protein kinase (MAPK) cascade [78]. Current theories assume that SHP2 acts as a scaffold protein binding GRB2 and SOS1, inducing an increase in nucleotide exchange. In the past, SHP2 has been considered to be expendable for KRAS^mut^ cancer types [79]. However, quite recently, a number of groups [80,81,82,83,84,85] have shown that SHP2 plays an integral role in the tumor progression, and even more so in the development of resistance mechanisms upon treatment with MEKi/ERKi. At the moment, a couple of SHP2i, amongst others TNO155 (Novartis: *NCT04330664*, *NCT03114319*) and RMC-4630 (Revolution Medicines: *NCT03989115*, *NCT04418661*), are being evaluated clinically in combination therapies in advanced solid tumors (Figure 1A and Table 2).

### 4.2. Disruption of Signaling Cascades Downstream of KRAS

The constitutive activation of the oncogenic KRAS protein induces continued stimulation of downstream signaling pathways leading, e.g., to uncontrolled proliferation, metabolic reprogramming, survival and increased migratory potential. Up to today, more than ten different effector families are known, the RAF-MEK-ERK MAPK cascade and the PI3K-AKT-mTOR cell survival pathway being the two best studied and most understood. Several inhibitors targeting the components of each of the two have been developed and are undergoing or have undergone clinical evaluation (Figure 1B).

Active KRAS preferentially interacts with RAF, thus, inducing its translocation to the plasma membrane and its phosphorylation. Here, RAF activates the dual specificity kinases MEK1/2. This leads to the activation via phosphorylation of ERK1/2 serine-threonine MAPKs. In turn, these can phosphorylate a broad spectrum of cytoplasmic and nuclear substrates. Currently, only three BRAF^mut^-selective inhibitors (specifically BRAF^V600E^ and BRAF^V600K^), namely, vemurafenib, dabrafenib and encorafenib, are clinically available for the treatment of BRAF^mut^ melanoma and/or BRAF^mut^ non-small cell lung cancer. All three inhibitors proved to be rather effective in BRAF^mut^ melanoma with response rates greater than 50% and major improvement in quality of life. Unexpectedly, in KRAS^mut^ cancers, including PDAC, either of these inhibitors induces a CRAF dimerization and unwanted activation of the RAF-MEK-ERK MAPK signaling cascade [86]. In contrast, novel pan-RAF inhibitors, such as LXH254, do not seem to trigger this phenomenon. Hence, they might be a valuable option for KRAS^mut^ cancer. Moreover, preclinical data suggest that inhibition of RAF may sensitize pancreatic tumors to the treatment with other targeted inhibitors [87,88,89]. Unfortunately, clinical data regarding the efficacy of pan-RAF inhibitors as mono or combination therapy in patients suffering from PDAC remain unavailable. When retracing the individual steps of the MAPK cascade, other appealing targets for the treatment of KRAS^mut^ cancer are the downstream effectors MEK1/2 and ERK1/2. Several MEK1/2 inhibitors, for example pimasertinib and trametinib, have reached clinical studies [90,91,92]. When the first was evaluated in combination with gemcitabine vs. gemcitabine alone as a first line therapy in metastatic pancreatic adenocarcinoma (*NCT01016483*), the combination treatment showed only slightly improved progression free survival (PFS), while inducing severe ocular side effects resulting overall in no clinical benefit [92]. When the latter was evaluated in a similar clinical set-up (trametinib with gemcitabine vs. gemcitabine; *NCT01231581*), it did not show a clinical benefit either. Hence, the clinical investigation of gemcitabine in combination with MEKi was abandoned [91]. These disappointing results can probably be attributed to the unleashing of pathway feedback loops in RAS mutant tumors upon MEKi leading to the modest activity observed in the clinic [93,94,95]. In the case of an acquired MEKi- or RAFi resistance, selective ERK inhibition appeared as a valuable treatment option [96,97,98,99]. However, in spite of promising preclinical data, the ERK inhibitors that have been tested in the clinic have fallen short of the expectations [58]. Nevertheless, in recent past years, several research groups have successfully focused their efforts on identifying viable treatment options based on MEKi or ERKi regimens in a (pre-) clinical setting (Table 2 and Table 3), e.g., the vertical combination of SHP2i with MEKi [83] or the disruption of increased autophagic flux in response to inhibition of MEK [100] or ERK [101]. Moreover, the results with MEKi and ERKi in vertical combination therapies in the treatment of other cancer entities with hyperactivation of the MAPK pathway have given rise to hope: vemurafenib in combination with cobimetinib, for example, has become an important tool in the treatment of BRAF^mut^ melanoma despite its side effects [102,103].

Just like the RAF-MEK-ERK mitogen-activated protein kinase (MAPK) cascade, the PI3K-AKT-mTOR pathway is involved in the regulation of essential cellular functions such as transcription, translation, proliferation, motility, metabolic adaptation and survival. Its aberrant activation contributes to the pathogenesis of several cancer entities, including PDAC [104,105]. PI3Ks fall into three classes (I–III), generally composed of heterodimers consisting of a regulatory and a catalytic subunit. Class I PI3Ks phosphorylate phosphatidylinositol 4,5-bisphosphate (PIP2) upon activation by RAS. In turn, PIP2 recruits AKT to the membrane and induces an activation of mTOR. More than 40 inhibitors of this survival pathway have reached different stages of clinical development, but only a few, e.g., temsirolimus (mTORi), everolimus (mTORi), idelalisib (PI3Ki), and copanlisib (PI3Ki), have been approved for clinical use. Additionally, none of these have been approved for the treatment of PDAC. The hurdles that led to the failure of the majority of PI3K-AKT-mTOR-targeting compounds in the clinic included limited single agent efficacy, dose-limiting toxicity and the lack of predictive biomarkers for patient stratification.

A combined inhibition of both MAPK and PI3K pathways showed favorable efficacy in preclinical models [106,107] as well as in an initial clinical trial [108], but at the expense of an increase in dose-limiting toxicity. Nevertheless, the obtained results hold great promise for a subset of patients, always considering the fact that the stratification based on genetic markers as well as adjustments of the regimen could lead to better tolerability while maintaining or even augmenting the efficacy of treatment.

## 5. Immune Modulatory Perspective

The microenvironment of PDAC is characterized by dysfunctional immune effector cells enhancing an immunosuppressive milieu. Multiple different cell types, amongst other cancer-associated fibroblast and macrophages as well as myeloid-derived suppressor cells, aid the immunosuppression. This is accomplished by both the excretion of certain cytokines and the expression of tolerance-inducing surface molecules in interplay with the tumor cells themselves: an initially anti-tumoral immune response is converted to a cancer-supportive microenvironment. Hence, a re-programming of the dysfunctional immune response in the treatment of PDAC holds great promise [109]. Currently, seven antibodies targeting three major immune check point proteins (anti-CTLA4, anit-PD1 and anti-PDL1) have been approved by the FDA for the treatment of several different tumor entities, including *KRAS^mut^* melanoma and non-small cell lung cancer [110]. Predictive factors for the efficacy of these treatments appear to be high expression levels of the respective target structures and increased numbers of tumor infiltrating lymphocytes (TILs) in combination with a high mutational burden of the tumor [111,112,113]. Unsurprisingly, the blockade of immune checkpoints on its own has disappointed in clinical trials in the treatment of PDAC, likely due to the predominant immunosuppressive milieu. However, KRAS^G12C^-selective inhibitors have shown the ability to harness the immune system to heighten therapeutic efficacy of check point inhibitors [53]. These results suggest additional mechanisms of immune evasion that are closely connected to mutant *KRAS*. Hence, the combination of KRASi with checkpoint inhibitors or other immunotherapeutic strategies is an attractive option. Currently, some of these combination therapies are undergoing clinical investigation (Table 1).

## 6. Conclusions

To further improve treatment outcome for patients suffering from PDAC, therapy will need to be precise and personalized. Based on a deepened understanding of the individual tumor and reliable and instant molecular stratification, patients would be allocated to receiving, e.g., cytotoxic chemotherapy, immunotherapy, molecular targeting of oncogenic signaling pathways, DNA damage response or epigenetic modifiers and eventually combinations thereof.

In this setting, one option would be a KRAS-targeting treatment. Unfortunately, no clinically approved KRAS-targeting treatment is available for pancreatic ductal adenocarcinoma as of yet. Though clinical data of targeted monotherapies have shown promising results in KRAS^mut^ cancer, they have been overshadowed by the swift development of resistance resulting in an inconsistent response. With the development of a new generation of RAS pathway-targeted therapies, especially the discovery of potent allele-specific KRASi, as well as the identification of resistance mechanisms, hope has been fueled to successfully treat KRAS^mut^ cancer, including PDAC, in a targeted fashion. It has become more and more evident that monotherapeutic targeting of KRAS is indeed futile. Nevertheless, preclinical data as well as clinical data from other tumor entities bearing *KRAS* mutations give rise to hope for promising combination therapies involving targeted inhibition of the RAS signaling pathway, paving the way for suitable treatment options for patients suffering from PDAC.

## Figures and Tables

**Table 1:** Compounds directly targeting mutated KRAS currently in clinical trials, which include patients suffering from PDAC; first steps towards a paradigm shift in the treatment of KRAS-dependent tumor entities.

Drug	Sponsor	Target	Binding Site	Phase	Indication	*n*=
**AMG-510** *NCT03600883*	Amgen	KRAS^G12C^	switch-II	I/II	solid tumors	533 ^a^
**AMG-510** *NCT04303780*	Amgen	KRAS^G12C^	switch-II	III	advanced mNSCLC	650 ^b^
**AMG-510** (with anti-PD1, MEKi, SHP2i, pan-ErbBi) *NCT04185883*	Amgen	KRAS^G12C^	switch-II	I	advanced solid tumors	430 ^a^
**MRTX849** (alone and with pembrolizumab, cetuximab or afatinib) *NCT03785249*	Mirati Therapeutics Inc.	KRAS^G12C^	switch-II	I/II	advanced solid tumors	200 ^a^
**MRTX849** (with TNO155) *NCT04330664*	Mirati Therapeutics Inc.	KRAS^G12C^	switch-II	I/II	advanced solid tumors	148 ^a^
**ARS-3248/** **JNJ-74699157** *NCT04006301*	Janssen	KRAS^G12C^	switch-II	I/II	advanced solid tumors (NSCLC; colon cancer)	10/140 ^c^
**LY3499446** (alone and with abemaciclib, cetuximab or erlotinib) *NCT04165031*	Eli Lilly and Company	KRAS^G12C^	switch-II	I/II	advanced solid tumors (NSCLC; colon cancer)	230 ^a^
**BI-1701963** (alone and with trametinib) *NCT04111458*	Boehringer Ingelheim	KRAS^mut^ (KRAS^wt^)	switch-I/II specifically SOS1:KRAS	I/II	solid tumors	140 ^a^

^a^ estimated enrollment numbers, recruitment has started; ^b^ estimated enrollment numbers, recruitment has not yet started; ^c^ not actively recruiting.

**Table 2:** Several compounds targeting the signaling cascade upstream of KRAS have reached clinical trials; however, only a few also include patients suffering from pancreatic ductal adenocarcinoma. Here, we focus on the inhibition of SHP2.

Drug	Sponsor	Target	Phase	Indication	*n*=
**RMC-4630** *NCT03634982*	Revolution Medicines, Inc.	SHP2	I	advanced solid tumors (with specific genotypic aberrations leading to RAS pathway hyperactivation)	240 ^a^
**RMC-4630** (with cobimetinib) *NCT03989115*	Revolution Medicines, Inc./Sanofi	SHP2	Ib/II	advanced solid tumors (with specific genotypic aberrations leading to RAS pathway hyperactivation)	144 ^a^
**RMC-4630** (with pembrolizumab) *NCT04418661*	Sanofi/Revolution Medicines, Inc.	SHP2	I	advanced solid tumors (with specific genotypic aberrations leading to RAS pathway hyperactivation)	24 ^a^
**TNO155** (with MRTX849) *NCT04330664*	Mirati Therapeutics Inc./Novartis	SHP2	I/II	advanced solid tumors (KRAS^G12C^)	148 ^a^
**JAB-3068** *NCT03565003*	Jacobio Pharmaceuticals Co., Ltd.	SHP2	I/IIa	advanced solid tumors	120 ^a^

^a^ estimated enrollment numbers, recruitment has started.

**Table 3:** Several compounds targeting the signaling cascade downstream of KRAS have reached clinical trials and were subsequently approved for clinical use. Here, we focus on ongoing major clinical trials that include advanced solid tumors or specifically pancreatic cancer.

Drug	Sponsor	Target	Phase	Indication	*n*=
**Belvarafenib** (with Cobimetinib) *NCT03284502*	Hanmi Pharmaceutical Company Limited	RAF	I	advanced solid tumors	272 ^a^
**LXH254** (alone and with Spartalizumab) *NCT02607813*	Novartis Pharmaceuticals	RAF	I	advanced solid tumors (with MAPK alterations)	152 ^a^
**RO5126766** (with VS-6063) *NCT03875820*	Institute of Cancer Research, UK/Verastem, Inc./Chugai Pharmaceutical	dual RAF/MEK	I	advanced RAS mutant solid tumors	80 ^a^
**RO5126766** (alone and with Everolismus) *NCT02407509*	Royal Marsden NHS Foundation Trust/Verastem, Inc./Chugai Pharmaceutical	dual RAF/MEK	I	solid tumors	94 ^a^
**Trametinib** (with Navitoclax) *NCT02079740*	National Cancer Institute (NCI)	MEK	I/II	Advanced solid tumors (incl. stage III/IV pancreatic cancer)	130 ^a^
**Trametinib** (with Hydroxychloroquine) *NCT03825289*	University of Utah/Novartis Pharmaceuticals	MEK	I	Advanced pancreatic cancer	33 ^a^
**Binimetinib** (with Avelumab and/or Talazoparib) *NCT03637491*	Pfizer	MEK	Ib/II	advanced solid tumors (with RAS-mutation) pancreatic cancer	122 ^a^
**MK-8353** (with Pembrolizumab) *NCT02972034*	Merck Sharp & Dohme Corp	ERK1/2	I	advanced solid tumors	96 ^a^
**JSI-1187** (alone and with Dabrafenib) *NCT04418167*	JS InnoPharm, LLC	ERK1/2	I	advanced solid tumors (with MAPK pathway mutations)	124 ^a^
**LY3214996** (alone or with Abemaciclib or Nab-Paclitaxel and Gemcitabine or Encorafenib and Cetuximab) *NCT02857270*	Eli Lilly and Company	ERK1/2	I	advanced solid tumors (incl. metastatic pancreatic ductal adenocarcinoma)	272 ^a^
**LY3214996** (alone and with Hydroxychloroquine) *NCT04386057*	Dana-Farber Cancer Institute/Eli Lilly and Company	ERK1/2	I	Pancreatic Cancer	52 ^a^
**Gedatolisib** (with Palbociclib) *NCT03065062*	Dana-Farber Cancer Institute/Pfizer	PI3K/mTOR	I	advanced solid tumors (incl. pancreatic cancer)	96 ^a^

^a^ estimated enrollment numbers, recruitment has started.

## References

[1] Chhoda A., Lu L., Clerkin B.M., Risch H., Farrell J.J. (2019). Current Approaches to Pancreatic Cancer Screening. Am. J. Pathol..

[2] Siegel R.L., Miller K.D., Jemal A. (2019). Cancer statistics, 2019. CA Cancer J. Clin..

[3] Rahib L., Smith B.D., Aizenberg R., Rosenzweig A.B., Fleshman J.M., Matrisian L.M. (2014). Projecting cancer incidence and deaths to 2030: The unexpected burden of thyroid, liver, and pancreas cancers in the United States. Cancer Res..

[4] Rawla P., Sunkara T., Gaduputi V. (2019). Epidemiology of Pancreatic Cancer: Global Trends, Etiology and Risk Factors. World. J. Oncol..

[5] Soreide K., Aagnes B., Moller B., Westgaard A., Bray F. (2010). Epidemiology of pancreatic cancer in Norway: Trends in incidence, basis of diagnosis and survival 1965–2007. Scand. J. Gastroenterol..

[6] Seufferlein T., Ettrich T.J. (2019). Treatment of pancreatic cancer-neoadjuvant treatment in resectable pancreatic cancer (PDAC). Transl. Gastroenterol. Hepatol..

[7] Chandana S., Babiker H.M., Mahadevan D. (2019). Therapeutic trends in pancreatic ductal adenocarcinoma (PDAC). Expert Opin. Investig. Drugs.

[8] Sanchez S.E., Trevino J.G. (2008). Current adjuvant and targeted therapies for pancreatic adenocarcinoma. Curr. Med. Chem..

[9] Binenbaum Y., Na’ara S., Gil Z. (2015). Gemcitabine resistance in pancreatic ductal adenocarcinoma. Drug. Resist. Updat..

[10] Coppola S., Carnevale I., Danen E.H.J., Peters G.J., Schmidt T., Assaraf Y.G., Giovannetti E. (2017). A mechanopharmacology approach to overcome chemoresistance in pancreatic cancer. Drug Resist. Updat..

[11] Dauer P., Nomura A., Saluja A., Banerjee S. (2017). Microenvironment in determining chemo-resistance in pancreatic cancer: Neighborhood matters. Pancreatology.

[12] Fujita H., Ohuchida K., Mizumoto K., Itaba S., Ito T., Nakata K., Yu J., Kayashima T., Souzaki R., Tajiri T. (2010). Gene expression levels as predictive markers of outcome in pancreatic cancer after gemcitabine-based adjuvant chemotherapy. Neoplasia.

[13] Grasso C., Jansen G., Giovannetti E. (2017). Drug resistance in pancreatic cancer: Impact of altered energy metabolism. Crit. Rev. Oncol. Hematol..

[14] Manji G.A., Olive K.P., Saenger Y.M., Oberstein P. (2017). Current and Emerging Therapies in Metastatic Pancreatic Cancer. Clin. Cancer Res..

[15] Nagathihalli N.S., Castellanos J.A., Shi C., Beesetty Y., Reyzer M.L., Caprioli R., Chen X., Walsh A.J., Skala M.C., Moses H.L. (2015). Signal Transducer and Activator of Transcription 3, Mediated Remodeling of the Tumor Microenvironment Results in Enhanced Tumor Drug Delivery in a Mouse Model of Pancreatic Cancer. Gastroenterology.

[16] Qin C., Yang G., Yang J., Ren B., Wang H., Chen G., Zhao F., You L., Wang W., Zhao Y. (2020). Metabolism of pancreatic cancer: Paving the way to better anticancer strategies. Mol. Cancer.

[17] Rajabpour A., Rajaei F., Teimoori-Toolabi L. (2017). Molecular alterations contributing to pancreatic cancer chemoresistance. Pancreatology.

[18] Aslan M., Shahbazi R., Ulubayram K., Ozpolat B. (2018). Targeted Therapies for Pancreatic Cancer and Hurdles Ahead. Anticancer Res..

[19] Aung K.L., Fischer S.E., Denroche R.E., Jang G.H., Dodd A., Creighton S., Southwood B., Liang S.B., Chadwick D., Zhang A. (2018). Genomics-Driven Precision Medicine for Advanced Pancreatic Cancer: Early Results from the COMPASS Trial. Clin. Cancer Res..

[20] Kelley R.K., Ko A.H. (2008). Erlotinib in the treatment of advanced pancreatic cancer. Biologics.

[21] Yang M., Shan B., Li Q., Song X., Cai J., Deng J., Zhang L., Du Z., Lu J., Chen T. (2013). Overcoming erlotinib resistance with tailored treatment regimen in patient-derived xenografts from naive Asian NSCLC patients. Int. J. Cancer.

[22] Salzberg M.P., Merchant N.B. (2017). Overcoming erlotinib resistance with STAT3 inhibition in pancreatic cancer. J. Clin. Oncol..

[23] Wong M.H., Xue A., Baxter R.C., Pavlakis N., Smith R.C. (2016). Upstream and Downstream Co-inhibition of Mitogen-Activated Protein Kinase and PI3K/Akt/mTOR Pathways in Pancreatic Ductal Adenocarcinoma. Neoplasia.

[24] Diep C.H., Munoz R.M., Choudhary A., Von Hoff D.D., Han H. (2011). Synergistic Effect between Erlotinib and MEK Inhibitors in KRAS Wild-Type Human Pancreatic Cancer Cells. Clin. Cancer Res..

[25] Fakhri B., Lim K.H. (2017). Molecular landscape and sub-classification of gastrointestinal cancers: A review of literature. J. Gastrointest. Oncol..

[26] Waddell N., Pajic M., Patch A.M., Chang D.K., Kassahn K.S., Bailey P., Johns A.L., Miller D., Nones K., Quek K. (2015). Whole genomes redefine the mutational landscape of pancreatic cancer. Nature.

[27] Wood L.D., Hruban R.H. (2015). Genomic landscapes of pancreatic neoplasia. J. Pathol. Transl. Med..

[28] Eser S., Schnieke A., Schneider G., Saur D. (2014). Oncogenic KRAS signalling in pancreatic cancer. Br. J. Cancer.

[29] Pylayeva-Gupta Y., Grabocka E., Bar-Sagi D. (2011). RAS oncogenes: Weaving a tumorigenic web. Nat. Rev. Cancer.

[30] Waters A.M., Der C.J. (2018). KRAS: The Critical Driver and Therapeutic Target for Pancreatic Cancer. Cold Spring Harb. Perspect. Med..

[31] Liu J., Ji S., Liang C., Qin Y., Jin K., Liang D., Xu W., Shi S., Zhang B., Liu L. (2016). Critical role of oncogenic KRAS in pancreatic cancer (Review). Mol. Med. Rep..

[32] Mueller S., Engleitner T., Maresch R., Zukowska M., Lange S., Kaltenbacher T., Konukiewitz B., Ollinger R., Zwiebel M., Strong A. (2018). Evolutionary routes and KRAS dosage define pancreatic cancer phenotypes. Nature.

[33] Raphael B.J., Hruban R.H., Aguirre A.J., Moffitt R.A., Yeh J.J., Stewart C., Robertson A.G., Cherniack A.D., Gupta M., Getz G. (2017). Integrated Genomic Characterization of Pancreatic Ductal Adenocarcinoma. Cancer Cell.

[34] Cox A.D., Fesik S.W., Kimmelman A.C., Luo J., Der C.J. (2014). Drugging the undruggable RAS: Mission possible?. Nat. Rev. Drug Discov..

[35] Liu P., Wang Y., Li X. (2019). Targeting the untargetable KRAS in cancer therapy. Acta Pharm. Sin. B.

[36] (2019). Race for undruggable KRAS speeds up. Nat. Biotechnol..

[37] Wright L.P., Philips M.R. (2006). Thematic review series: Lipid posttranslational modifications. CAAX modification and membrane targeting of Ras. J. Lipid Res..

[38] Berndt N., Hamilton A.D., Sebti S.M. (2011). Targeting protein prenylation for cancer therapy. Nat. Rev. Cancer.

[39] Basso A.D., Kirschmeier P., Bishop W.R. (2006). Lipid posttranslational modifications. Farnesyl transferase inhibitors. J. Lipid Res..

[40] Fleming J.B., Shen G.L., Holloway S.E., Davis M., Brekken R.A. (2005). Molecular consequences of silencing mutant K-ras in pancreatic cancer cells: Justification for K-ras-directed therapy. Mol. Cancer Res..

[41] Gu L., Deng Z.J., Roy S., Hammond P.T. (2017). A Combination RNAi-Chemotherapy Layer-by-Layer Nanoparticle for Systemic Targeting of KRAS/P53 with Cisplatin to Treat Non-Small Cell Lung Cancer. Clin. Cancer Res..

[42] Lim K.H., Counter C.M. (2005). Reduction in the requirement of oncogenic Ras signaling to activation of PI3K/AKT pathway during tumor maintenance. Cancer Cell.

[43] Pecot C.V., Wu S.Y., Bellister S., Filant J., Rupaimoole R., Hisamatsu T., Bhattacharya R., Maharaj A., Azam S., Rodriguez-Aguayo C. (2014). Therapeutic silencing of KRAS using systemically delivered siRNAs. Mol. Cancer.

[44] Strand M.S., Krasnick B.A., Pan H., Zhang X., Bi Y., Brooks C., Wetzel C., Sankpal N., Fleming T., Goedegebuure S.P. (2019). Precision delivery of RAS-inhibiting siRNA to KRAS driven cancer via peptide-based nanoparticles. Oncotarget.

[45] Xue W., Dahlman J.E., Tammela T., Khan O.F., Sood S., Dave A., Cai W., Chirino L.M., Yang G.R., Bronson R. (2014). Small RNA combination therapy for lung cancer. Proc. Natl. Acad. Sci. USA.

[46] Yuan T.L., Fellmann C., Lee C.S., Ritchie C.D., Thapar V., Lee L.C., Hsu D.J., Grace D., Carver J.O., Zuber J. (2014). Development of siRNA payloads to target KRAS-mutant cancer. Cancer Discov..

[47] Golan T., Khvalevsky E.Z., Hubert A., Gabai R.M., Hen N., Segal A., Domb A., Harari G., David E.B., Raskin S. (2015). RNAi therapy targeting KRAS in combination with chemotherapy for locally advanced pancreatic cancer patients. Oncotarget.

[48] Ostrem J.M., Peters U., Sos M.L., Wells J.A., Shokat K.M. (2013). K-Ras(G12C) inhibitors allosterically control GTP affinity and effector interactions. Nature.

[49] Patricelli M.P., Janes M.R., Li L.S., Hansen R., Peters U., Kessler L.V., Chen Y., Kucharski J.M., Feng J., Ely T. (2016). Selective Inhibition of Oncogenic KRAS Output with Small Molecules Targeting the Inactive State. Cancer Discov..

[50] Hansen R., Peters U., Babbar A., Chen Y., Feng J., Janes M.R., Li L.S., Ren P., Liu Y., Zarrinkar P.P. (2018). The reactivity-driven biochemical mechanism of covalent KRAS(G12C) inhibitors. Nat. Struct. Mol. Biol..

[51] Hobbs G.A., Wittinghofer A., Der C.J. (2016). Selective Targeting of the KRAS G12C Mutant: Kicking KRAS When It’s Down. Cancer Cell.

[52] Fakih M., O’Neil B., Price T.J., Falchook G.S., Desai J., Kuo J., Govindan R., Rasmussen E., Morrow P.K.H., Ngang J. (2019). Phase 1 study evaluating the safety, tolerability, pharmacokinetics (PK), and efficacy of AMG 510, a novel small molecule KRAS(G12c) inhibitor, in advanced solid tumors. J. Clin. Oncol..

[53] Canon J., Rex K., Saiki A.Y., Mohr C., Cooke K., Bagal D., Gaida K., Holt T., Knutson C.G., Koppada N. (2019). The clinical KRAS(G12C) inhibitor AMG 510 drives anti-tumour immunity. Nature.

[54] Hallin J., Engstrom L.D., Hargis L., Calinisan A., Aranda R., Briere D.M., Sudhakar N., Bowcut V., Baer B.R., Ballard J.A. (2020). The KRAS(G12C) Inhibitor MRTX849 Provides Insight toward Therapeutic Susceptibility of KRAS-Mutant Cancers in Mouse Models and Patients. Cancer Discov..

[55] Hunter J.C., Manandhar A., Carrasco M.A., Gurbani D., Gondi S., Westover K.D. (2015). Biochemical and Structural Analysis of Common Cancer-Associated KRAS Mutations. Mol. Cancer Res..

[56] Haigis K.M. (2017). KRAS Alleles: The Devil Is in the Detail. Trends Cancer.

[57] Hobbs G.A., Baker N.M., Miermont A.M., Thurman R.D., Pierobon M., Tran T.H., Anderson A.O., Waters A.M., Diehl J.N., Papke B. (2020). Atypical KRAS(G12R) Mutant Is Impaired in PI3K Signaling and Macropinocytosis in Pancreatic Cancer. Cancer Discov..

[58] Moore A.R., Rosenberg S.C., McCormick F., Malek S. (2020). RAS-targeted therapies: Is the undruggable drugged?. Nat. Rev. Drug Discov..

[59] Kessler D., Gmachl M., Mantoulidis A., Martin L.J., Zoephel A., Mayer M., Gollner A., Covini D., Fischer S., Gerstberger T. (2019). Drugging an undruggable pocket on KRAS. Proc. Natl. Acad. Sci. USA.

[60] Nickerson S., Joy S.T., Arora P.S., Bar-Sagi D., Tamanoi F., Der C.J. (2013). An orthosteric inhibitor of the RAS-SOS interaction. The Enzymes.

[61] Mattox T.E., Chen X., Maxuitenko Y.Y., Keeton A.B., Piazza G.A. (2019). Exploiting RAS Nucleotide Cycling as a Strategy for Drugging RAS-Driven Cancers. Int. J. Mol. Sci..

[62] Cannataro V.L., Gaffney S.G., Stender C., Zhao Z.M., Philips M., Greenstein A.E., Townsend J.P. (2018). Heterogeneity and mutation in KRAS and associated oncogenes: Evaluating the potential for the evolution of resistance to targeting of KRAS G12C. Oncogene.

[63] Xue J.Y., Zhao Y., Aronowitz J., Mai T.T., Vides A., Qeriqi B., Kim D., Li C., de Stanchina E., Mazutis L. (2020). Rapid non-uniform adaptation to conformation-specific KRAS(G12C) inhibition. Nature.

[64] Kapoor A., Yao W., Ying H., Hua S., Liewen A., Wang Q., Zhong Y., Wu C.-J., Sadanandam A., Hu B. (2014). Yap1 Activation Enables Bypass of Oncogenic Kras Addiction in Pancreatic Cancer. Cell.

[65] Viale A., Pettazzoni P., Lyssiotis C.A., Ying H., Sánchez N., Marchesini M., Carugo A., Green T., Seth S., Giuliani V. (2014). Oncogene ablation-resistant pancreatic cancer cells depend on mitochondrial function. Nature.

[66] Ying H., Kimmelman A.C., Lyssiotis C.A., Hua S., Chu G.C., Fletcher-Sananikone E., Locasale J.W., Son J., Zhang H., Coloff J.L. (2012). Oncogenic Kras maintains pancreatic tumors through regulation of anabolic glucose metabolism. Cell.

[67] Vaseva A.V., Blake D.R., Gilbert T.S.K., Ng S., Hostetter G., Azam S.H., Ozkan-Dagliyan I., Gautam P., Bryant K.L., Pearce K.H. (2018). KRAS Suppression-Induced Degradation of MYC Is Antagonized by a MEK5-ERK5 Compensatory Mechanism. Cancer Cell.

[68] Ryan M.B., Fece de la Cruz F., Phat S., Myers D.T., Wong E., Shahzade H.A., Hong C.B., Corcoran R.B. (2020). Vertical Pathway Inhibition Overcomes Adaptive Feedback Resistance to KRAS(G12C) Inhibition. Clin. Cancer Res..

[69] Misale S., Fatherree J.P., Cortez E., Li C., Bilton S., Timonina D., Myers D.T., Lee D., Gomez-Caraballo M., Greenberg M. (2019). KRAS G12C NSCLC Models Are Sensitive to Direct Targeting of KRAS in Combination with PI3K Inhibition. Clin. Cancer Res..

[70] Young A., Lyons J., Miller A.L., Phan V.T., Alarcon I.R., McCormick F. (2009). Ras signaling and therapies. Adv. Cancer Res..

[71] Omerovic J., Laude A.J., Prior I.A. (2007). Ras proteins: Paradigms for compartmentalised and isoform-specific signalling. Cell Mol. Life Sci..

[72] Ardito C.M., Gruner B.M., Takeuchi K.K., Lubeseder-Martellato C., Teichmann N., Mazur P.K., Delgiorno K.E., Carpenter E.S., Halbrook C.J., Hall J.C. (2012). EGF receptor is required for KRAS-induced pancreatic tumorigenesis. Cancer Cell.

[73] Mazur P.K., Siveke J.T. (2012). Genetically engineered mouse models of pancreatic cancer: Unravelling tumour biology and progressing translational oncology. Gut.

[74] Boeck S., Jung A., Laubender R.P., Neumann J., Egg R., Goritschan C., Ormanns S., Haas M., Modest D.P., Kirchner T. (2013). KRAS mutation status is not predictive for objective response to anti-EGFR treatment with erlotinib in patients with advanced pancreatic cancer. J. Gastroenterol..

[75] Sun Q., Burke J.P., Phan J., Burns M.C., Olejniczak E.T., Waterson A.G., Lee T., Rossanese O.W., Fesik S.W. (2012). Discovery of small molecules that bind to K-Ras and inhibit Sos-mediated activation. Angew. Chem. Int. Ed. Engl..

[76] Patgiri A., Yadav K.K., Arora P.S., Bar-Sagi D. (2011). An orthosteric inhibitor of the Ras-Sos interaction. Nat. Chem. Biol..

[77] Leshchiner E.S., Parkhitko A., Bird G.H., Luccarelli J., Bellairs J.A., Escudero S., Opoku-Nsiah K., Godes M., Perrimon N., Walensky L.D. (2015). Direct inhibition of oncogenic KRAS by hydrocarbon-stapled SOS1 helices. Proc. Natl. Acad. Sci. USA.

[78] Shi Z.Q., Yu D.H., Park M., Marshall M., Feng G.S. (2000). Molecular mechanism for the Shp-2 tyrosine phosphatase function in promoting growth factor stimulation of Erk activity. Mol. Cell. Biol..

[79] Chen Y.N., LaMarche M.J., Chan H.M., Fekkes P., Garcia-Fortanet J., Acker M.G., Antonakos B., Chen C.H., Chen Z., Cooke V.G. (2016). Allosteric inhibition of SHP2 phosphatase inhibits cancers driven by receptor tyrosine kinases. Nature.

[80] Wong G.S., Zhou J., Liu J.B., Wu Z., Xu X., Li T., Xu D., Schumacher S.E., Puschhof J., McFarland J. (2018). Targeting wild-type KRAS-amplified gastroesophageal cancer through combined MEK and SHP2 inhibition. Nat. Med..

[81] Nichols R.J., Haderk F., Stahlhut C., Schulze C.J., Hemmati G., Wildes D., Tzitzilonis C., Mordec K., Marquez A., Romero J. (2018). RAS nucleotide cycling underlies the SHP2 phosphatase dependence of mutant BRAF-, NF1- and RAS-driven cancers. Nat. Cell Biol..

[82] Dardaei L., Wang H.Q., Singh M., Fordjour P., Shaw K.X., Yoda S., Kerr G., Yu K., Liang J., Cao Y. (2018). SHP2 inhibition restores sensitivity in ALK-rearranged non-small-cell lung cancer resistant to ALK inhibitors. Nat. Med..

[83] Ruess D.A., Heynen G.J., Ciecielski K.J., Ai J., Berninger A., Kabacaoglu D., Gorgulu K., Dantes Z., Wormann S.M., Diakopoulos K.N. (2018). Mutant KRAS-driven cancers depend on PTPN11/SHP2 phosphatase. Nat. Med..

[84] Fedele C., Ran H., Diskin B., Wei W., Jen J., Geer M.J., Araki K., Ozerdem U., Simeone D.M., Miller G. (2018). SHP2 Inhibition Prevents Adaptive Resistance to MEK Inhibitors in Multiple Cancer Models. Cancer Discov..

[85] Ahmed T.A., Adamopoulos C., Karoulia Z., Wu X., Sachidanandam R., Aaronson S.A., Poulikakos P.I. (2019). SHP2 Drives Adaptive Resistance to ERK Signaling Inhibition in Molecularly Defined Subsets of ERK-Dependent Tumors. Cell Rep..

[86] Lito P., Rosen N., Solit D.B. (2013). Tumor adaptation and resistance to RAF inhibitors. Nat. Med..

[87] Chen S.H., Gong X., Zhang Y., Van Horn R.D., Yin T., Huber L., Burke T.F., Manro J., Iversen P.W., Wu W. (2018). RAF inhibitor LY3009120 sensitizes RAS or BRAF mutant cancer to CDK4/6 inhibition by abemaciclib via superior inhibition of phospho-RB and suppression of cyclin D1. Oncogene.

[88] Del Curatolo A., Conciatori F., Cesta Incani U., Bazzichetto C., Falcone I., Corbo V., D’Agosto S., Eramo A., Sette G., Sperduti I. (2018). Therapeutic potential of combined BRAF/MEK blockade in BRAF-wild type preclinical tumor models. J. Exp. Clin. Cancer Res..

[89] Blasco M.T., Navas C., Martin-Serrano G., Grana-Castro O., Lechuga C.G., Martin-Diaz L., Djurec M., Li J., Morales-Cacho L., Esteban-Burgos L. (2019). Complete Regression of Advanced Pancreatic Ductal Adenocarcinomas upon Combined Inhibition of EGFR and C-RAF. Cancer Cell.

[90] Goncalves A., Gilabert M., Francois E., Dahan L., Perrier H., Lamy R., Re D., Largillier R., Gasmi M., Tchiknavorian X. (2012). BAYPAN study: A double-blind phase III randomized trial comparing gemcitabine plus sorafenib and gemcitabine plus placebo in patients with advanced pancreatic cancer. Ann. Oncol..

[91] Infante J.R., Somer B.G., Park J.O., Li C.P., Scheulen M.E., Kasubhai S.M., Oh D.Y., Liu Y., Redhu S., Steplewski K. (2014). A randomised, double-blind, placebo-controlled trial of trametinib, an oral MEK inhibitor, in combination with gemcitabine for patients with untreated metastatic adenocarcinoma of the pancreas. Eur. J. Cancer.

[92] Van Cutsem E., Hidalgo M., Canon J.L., Macarulla T., Bazin I., Poddubskaya E., Manojlovic N., Radenkovic D., Verslype C., Raymond E. (2018). Phase I/II trial of pimasertib plus gemcitabine in patients with metastatic pancreatic cancer. Int. J. Cancer.

[93] Friday B.B., Yu C.R., Dy G.K., Smith P.D., Wang L., Thibodeau S.N., Adjei A.A. (2008). BRAF V600E disrupts AZD6244-Induced abrogation of negative feedback pathways between extracellular signal-regulated kinase and Raf proteins. Cancer Res..

[94] Hatzivassiliou G., Haling J.R., Chen H.F., Song K., Price S., Heald R., Hewitt J.F.M., Zak M., Peck A., Orr C. (2013). Mechanism of MEK inhibition determines efficacy in mutant KRAS- versus BRAF-driven cancers. Nature.

[95] Pratilas C.A., Taylor B.S., Ye Q., Viale A., Sander C., Solit D.B., Rosen N. (2009). (V600E)BRAF is associated with disabled feedback inhibition of RAF-MEK signaling and elevated transcriptional output of the pathway. Proc. Natl. Acad. Sci. USA.

[96] Hayes T.K., Neel N.F., Hu C., Gautam P., Chenard M., Long B., Aziz M., Kassner M., Bryant K.L., Pierobon M. (2016). Long-Term ERK Inhibition in KRAS-Mutant Pancreatic Cancer Is Associated with MYC Degradation and Senescence-like Growth Suppression. Cancer Cell.

[97] Ozkan-Dagliyan I., Diehl J.N., George S.D., Schaefer A., Papke B., Klotz-Noack K., Waters A.M., Goodwin C.M., Gautam P., Pierobon M. (2020). Low-Dose Vertical Inhibition of the RAF-MEK-ERK Cascade Causes Apoptotic Death of KRAS Mutant Cancers. Cell Rep..

[98] Hatzivassiliou G., Liu B.N., O’Brien C., Spoerke J.M., Hoeflich K.P., Haverty P.M., Soriano R., Forrest W.F., Heldens S., Chen H. (2012). ERK Inhibition Overcomes Acquired Resistance to MEK Inhibitors. Mol. Cancer Ther..

[99] Morris E.J., Jha S., Restaino C.R., Dayananth P., Zhu H., Cooper A., Carr D., Deng Y.G., Jin W.H., Black S. (2013). Discovery of a Novel ERK Inhibitor with Activity in Models of Acquired Resistance to BRAF and MEK Inhibitors. Cancer Discov..

[100] Kinsey C.G., Camolotto S.A., Boespflug A.M., Guillen K.P., Foth M., Truong A., Schuman S.S., Shea J.E., Seipp M.T., Yap J.T. (2019). Protective autophagy elicited by RAF-->MEK-->ERK inhibition suggests a treatment strategy for RAS-driven cancers. Nat. Med..

[101] Bryant K.L., Stalnecker C.A., Zeitouni D., Klomp J.E., Peng S., Tikunov A.P., Gunda V., Pierobon M., Waters A.M., George S.D. (2019). Combination of ERK and autophagy inhibition as a treatment approach for pancreatic cancer. Nat. Med..

[102] Sullivan R.J., Hamid O., Gonzalez R., Infante J.R., Patel M.R., Hodi F.S., Lewis K.D., Tawbi H.A., Hernandez G., Wongchenko M.J. (2019). Atezolizumab plus cobimetinib and vemurafenib in BRAF-mutated melanoma patients. Nat. Med..

[103] Wongchenko M.J., McArthur G.A., Dreno B., Larkin J., Ascierto P.A., Sosman J., Andries L., Kockx M., Hurst S.D., Caro I. (2017). Gene Expression Profiling in BRAF-Mutated Melanoma Reveals Patient Subgroups with Poor Outcomes to Vemurafenib That May Be Overcome by Cobimetinib Plus Vemurafenib. Clin. Cancer Res..

[104] Ebrahimi S., Hosseini M., Shahidsales S., Maftouh M., Ferns G.A., Ghayour-Mobarhan M., Hassanian S.M., Avan A. (2017). Targeting the Akt/PI3K Signaling Pathway as a Potential Therapeutic Strategy for the Treatment of Pancreatic Cancer. Curr. Med. Chem..

[105] Milton C.K., Self A.J., Clarke P.A., Banerji U., Piccioni F., Root D.E., Whittaker S.R. (2020). A genome-scale CRISPR screen identifies the ERBB and mTOR signalling networks as key determinants of response to PI3K inhibition in pancreatic cancer. Mol. Cancer Ther..

[106] Jiang H.M., Xu M., Li L., Highkin M., Zhang D.X., Li Q., Wang-Gillam A., Lim K.H. (2018). Concurrent HER or PI3K inhibition potentiates the anti-tumor effect of ERK inhibitor BVD-523 (ulixertinib) in preclinical pancreatic cancer models. Cancer Res..

[107] Nagathihalli N.S., Castellanos J.A., Lamichhane P., Messaggio F., Shi C.J., Dai X.Z., Rai P., Chen X., VanSaun M.N., Merchant N.B. (2018). Inverse Correlation of STAT3 and MEK Signaling Mediates Resistance to RAS Pathway Inhibition in Pancreatic Cancer. Cancer Res..

[108] Shimizu T., Tolcher A.W., Papadopoulos K.P., Beeram M., Rasco D.W., Smith L.S., Gunn S., Smetzer L., Mays T.A., Kaiser B. (2012). The Clinical Effect of the Dual-Targeting Strategy Involving PI3K/AKT/mTOR and RAS/MEK/ERK Pathways in Patients with Advanced Cancer. Clin. Cancer Res..

[109] Brunner M., Wu Z., Krautz C., Pilarsky C., Grutzmann R., Weber G.F. (2019). Current Clinical Strategies of Pancreatic Cancer Treatment and Open Molecular Questions. Int. J. Mol. Sci..

[110] Vaddepally R.K., Kharel P., Pandey R., Garje R., Chandra A.B. (2020). Review of Indications of FDA-Approved Immune Checkpoint Inhibitors per NCCN Guidelines with the Level of Evidence. Cancers.

[111] Van Allen E.M., Miao D., Schilling B., Shukla S.A., Blank C., Zimmer L., Sucker A., Hillen U., Foppen M.H.G., Goldinger S.M. (2015). Genomic correlates of response to CTLA-4 blockade in metastatic melanoma. Science.

[112] Keenan T.E., Burke K.P., Van Allen E.M. (2019). Genomic correlates of response to immune checkpoint blockade. Nat. Med..

[113] Topalian S.L., Hodi F.S., Brahmer J.R., Gettinger S.N., Smith D.C., McDermott D.F., Powderly J.D., Carvajal R.D., Sosman J.A., Atkins M.B. (2012). Safety, Activity, and Immune Correlates of Anti-PD-1 Antibody in Cancer. N. Engl. J. Med..

